# A New Cosmeceutical Approach for Mature Skin: Antioxidant Activity, In Vitro Permeation, and Preliminary Clinical Efficacy of Melatonin–N-Acetylcysteine Creams

**DOI:** 10.3390/antiox15080991

**Published:** 2026-08-10

**Authors:** Magdalena Bîrsan, Ruxandra Ștefănescu, Daniela-Lucia Muntean, Robert-Alexandru Vlad, Alin-Viorel Focșa, Cristina-Elena Iancu, Șadiye-Ioana Scripcariu, Adriana Ciurba

**Affiliations:** 1 Grigore T. Popa University of Medicine and Pharmacy Iasi, 16 Universitatii Street, 700115 Iasi, Romania; 2 Department of Pharmacognosy and Phytotherapy, Faculty of Pharmacy, George Emil Palade University of Medicine, Pharmacy, Science and Technology of Targu Mures, 38 Gheorghe Marinescu Street, 540142 Targu Mures, Romania; 3 Department of Analytical Chemistry, George Emil Palade University of Medicine, Pharmacy, Science and Technology of Targu Mures, 540142 Targu Mures, Romania; 4 Pharmaceutical Technology and Cosmetology Department, Faculty of Pharmacy, George Emil Palade University of Medicine, Pharmacy, Science and Technology of Targu Mures, 38th Gheorghe Marinescu Street, 540142 Targu Mures, Romania

**Keywords:** melatonin, *N*-acetylcysteine, antioxidants, cosmeceuticals, skin permeation, anti-aging, oxidative stress, topical formulations, Franz diffusion cells, HPLC

## Abstract

Skin ageing is associated with oxidative stress, mitochondrial dysfunction, extracellular matrix alterations, and impaired barrier function, supporting interest in antioxidant cosmeceuticals for mature skin. This study investigated the antioxidant activity of melatonin and *N*-acetylcysteine, the in vitro permeation of melatonin from different cream formulations, and their preliminary clinical performance in women aged 55–65 years. Antioxidant activity was assessed using 2,2-diphenyl-1-picrylhydrazyl (DPPH) and 2,2′-azino-bis(3-ethylbenzothiazoline-6-sulfonic acid)/Trolox equivalent antioxidant capacity (ABTS/TEAC) assays. Melatonin permeation across Strat-M^®^ membranes was evaluated using Franz diffusion cells and quantified by a validated high-performance liquid chromatography method coupled with a photodiode-array detection (HPLC-PDA) method. Clinical changes were assessed over 28 days using the Glogau wrinkle scale and a lower facial ptosis scale. *N*-acetylcysteine exhibited greater antioxidant activity than melatonin in the DPPH assay, with half-maximal inhibitory concentration (IC_50_) values of 5.96 ± 1.02 and 530.11 ± 48.63 µg/mL, respectively. Melatonin permeation varied according to formulation composition, with cumulative amounts after 24 h ranging from 81.27 ± 17.47 to 1043.48 ± 33.43 µg/cm^2^. Reductions in periocular wrinkle and lower facial ptosis scores were observed after 28 days. These preliminary findings support further investigation of melatonin–*N*-acetylcysteine creams for mature skin. However, the present membrane model did not assess cutaneous retention, and the clinical observations require confirmation in larger, controlled studies.

## 1. Introduction

Skin ageing is a complex biological process characterised by progressive changes in epidermal structure, collagen and elastin synthesis, barrier function, hydration, and transepidermal water loss [1,2,3,4,5]. These alterations become increasingly evident after 50–55 years of age as a result of hormonal changes, cumulative environmental exposure, mitochondrial dysfunction, and reduced regenerative capacity [6,7,8]. Oxidative stress contributes to this process through lipid peroxidation, damage to DNA and proteins, persistent low-grade inflammation, and degradation of extracellular matrix components [5,6,7]. These changes provide a rationale for investigating topical antioxidants in the care of mature skin.

**Figure 1:**
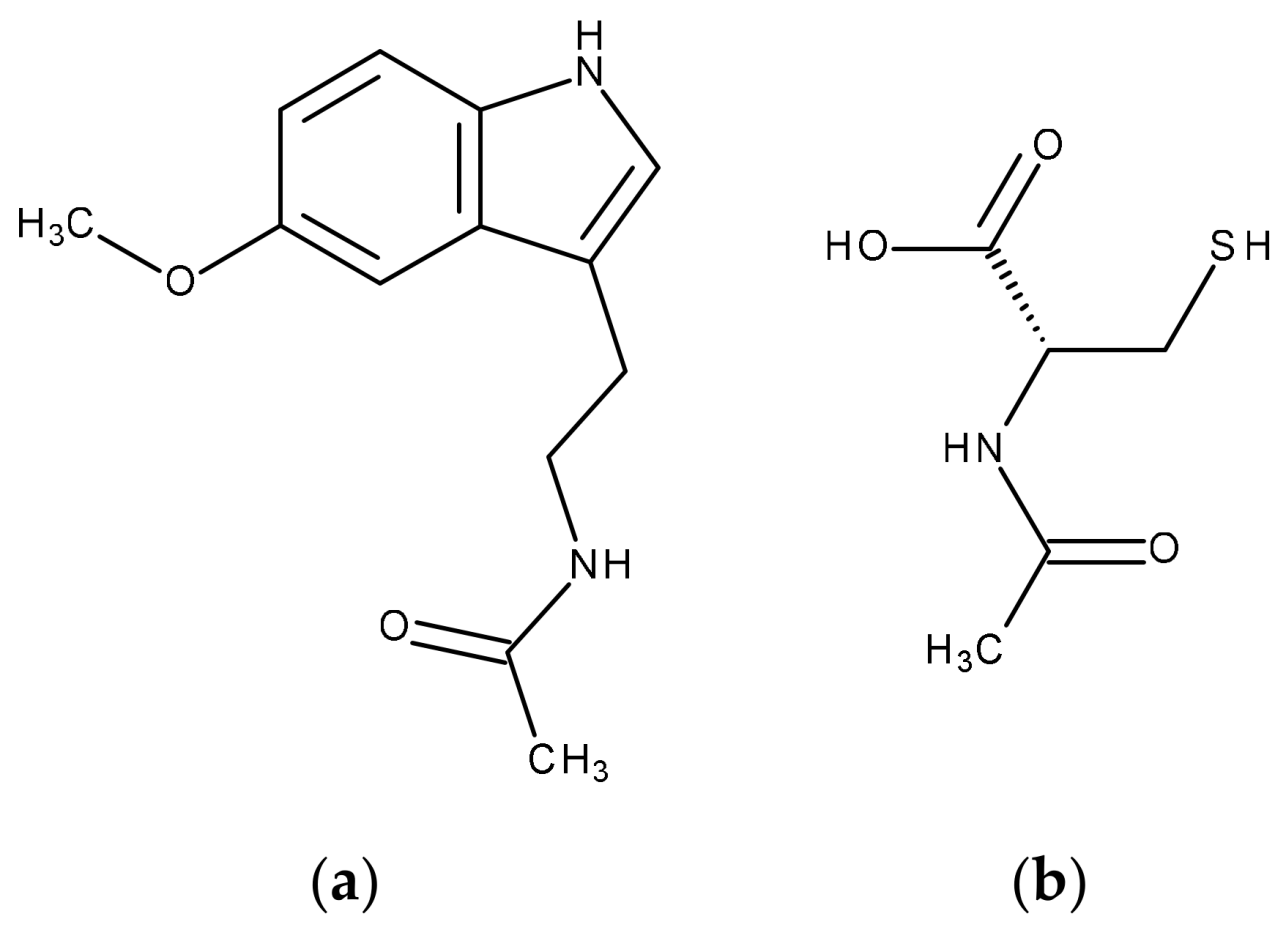
Chemical structures of (**a**) melatonin and (**b**) *N*-acetylcysteine.

Melatonin (Figure 1a) has attracted interest in cutaneous research because of its direct and indirect antioxidant properties [9,10,11,12,13,14,15]. Its electron-rich indole structure supports the scavenging of reactive oxygen and nitrogen species, while its metabolites contribute to a sequential antioxidant cascade [10,11,12]. Experimental and clinical studies have associated topical melatonin with photoprotective effects and improvements in skin hydration, elasticity, roughness, and overall appearance [10,11,12,15]. Mitochondrial protection and reduced oxidative damage to collagen and elastin have been proposed as possible mechanisms, although these effects were not investigated directly in the present study.

*N*-acetylcysteine (Figure 1b) is a thiol-containing compound and a precursor of glutathione with recognised antioxidant and redox-modulating properties [13,14]. Its free thiol group contributes to direct chemical reactivity, while cellular studies have described effects on intracellular glutathione availability and redox homeostasis. These properties may complement the antioxidant mechanisms reported for melatonin [10,13,14]. Nevertheless, the biological interaction between the two compounds and any additive or synergistic effects require direct experimental confirmation.

The commercial use of these ingredients also indicates increasing interest in their incorporation into skincare products. Melatonin is present in products such as ISDIN Melatonik^®^ Night Serum, Zelens Z Melatonin Night Cream, and MAXCLINIC Time Return Melatonin Cream, whereas *N*-acetylcysteine is included in formulations such as 111SKIN Repair Serum NAC Y^2TM^. These examples illustrate the commercial development of topical products containing either melatonin or *N*-acetylcysteine; however, their market availability does not constitute evidence of clinical efficacy. Published information concerning formulations that combine both compounds for mature skin remains limited.

The performance of topical antioxidants depends not only on the properties of the active ingredients but also on the composition and microstructure of the vehicle. Emulsifying systems, excipient selection, and physicochemical interactions within the formulation can influence active-substance release and permeation [16,17,18,19,20,21]. Franz diffusion cells provide a reproducible method for comparing permeation across synthetic or biological membranes. However, experiments using a Strat-M^®^ synthetic membrane measure transmembrane transport and cannot determine deposition within the stratum corneum, viable epidermis, or dermis. Direct assessment of cutaneous retention requires biological skin models and separate analysis of individual tissue compartments.

Previous studies have shown that vehicle architecture and ingredient compatibility can affect the stability and permeation behaviour of topical formulations [19,20,21,22,23,24,25]. Comparing cream bases prepared with different emulsifying systems may therefore provide relevant information on formulation-dependent melatonin transport. The combined use of chemical antioxidant assays, membrane-permeation testing, and clinical scoring offers complementary information during the initial evaluation of multi-active topical formulations, provided that the limitations of each method are clearly recognised.

Accordingly, the present study aimed to evaluate the chemical radical-scavenging capacity of melatonin and *N*-acetylcysteine using the DPPH and ABTS/TEAC assays, compare the in vitro permeation of melatonin from different cream formulations using Franz diffusion cells and Strat-M^®^ membranes, and conduct a preliminary 28-day clinical evaluation of selected formulations in women aged 55–65 years. Cutaneous retention, intracellular antioxidant mechanisms, and biological markers of skin ageing were not directly assessed.

## 2. Materials and Methods

### 2.1. Chemicals and Reagents

Melatonin was obtained from Fagron (Trikala-Larissa, Greece) and Cactus Botanics (City of Industry, CA, USA; purity 99%, used as analytical reference standard). *N*-acetylcysteine, arginine hydrochloride, low-molecular-weight hyaluronic acid, Aloe barbadensis leaf juice, Cocos nucifera oil, Argania spinosa kernel oil, and Centaurea cyanus flower water were purchased from Fagron (Trikala-Larissa, Greece). Blainvillea acmella flower extract was obtained from Select Botanical (Barcelona, Spain). α-Tocopherol, stearic acid, cetyl alcohol, and cetyl palmitate were purchased from Medchim TM (Bucharest, Romania). Euxyl^®^ PE 9010 was supplied by Dow Chemical (Midland, MI, USA). Methyl glucose sesquistearate and Ceteareth^®^-20 were obtained from Lehvoss (Origgio, Italy), while polyglyceryl-3 methylglucose distearate was obtained from Evonik (Essen, Germany).

DPPH was purchased from Sigma-Aldrich (Steinheim, Germany). ABTS was obtained from Roche Diagnostics GmbH (Mannheim, Germany), and potassium persulfate was purchased from Sigma-Aldrich (St. Louis, MO, USA). HPLC-grade acetonitrile was supplied by SLW Chemicals (Muskegon, MI, USA). All other reagents and solvents were of analytical grade.

### 2.2. DPPH Radical Scavenging Assay

The antioxidant activity of melatonin and *N*-acetylcysteine was evaluated using the DPPH radical-scavenging assay, with ascorbic acid as the reference antioxidant standard [26,27].

Stock solutions of *N*-acetylcysteine (0.05 g/5 mL), melatonin (0.01 g/5 mL), and DPPH solution (0.1 mM) were prepared before analysis. The samples were added to 96-well microplates and serially diluted using two-fold dilutions. Subsequently, 100 µL of DPPH solution was added to each well [27,28]. The plates were incubated for 30 min at room temperature in the dark.

The method is based on the reduction of the violet-colored stable DPPH radical to the yellow-colored diphenylpicrylhydrazine form in the presence of antioxidant compounds [28,29]. Absorbance was measured at 517 nm using an Epoch Microplate Spectrophotometer (BioTek Instruments Inc., Winooski, VT, USA).

The DPPH radical inhibition percentage was calculated according to the following equation:

Inhibition (%) = [(A0 − A1)/A0] × 100(1)

where A0 represents the absorbance of the DPPH solution and A1 represents the absorbance of the tested sample measured 30 min after DPPH addition.

All measurements were performed in triplicate, and the IC_50_ values were calculated by linear regression.

### 2.3. ABTS Radical Cation Decolorization Assay and TEAC Determination

The antioxidant activity of melatonin and *N*-acetylcysteine was further evaluated using the ABTS radical cation assay, expressed as Trolox Equivalent Antioxidant Capacity (TEAC). Trolox, a water-soluble vitamin E analogue, was used as the reference antioxidant standard.

Stock solutions of *N*-acetylcysteine (0.05 g/5 mL) and melatonin (0.01 g/5 mL) were prepared. The ABTS radical cation was generated by reacting 7 mM ABTS aqueous solution with 2.45 mM potassium persulfate. The mixture was kept in the dark at room temperature for 16–24 h to allow complete formation of ABTS•+.

Before use, the ABTS•+ stock solution was diluted with methanol to obtain an absorbance of 0.700 ± 0.020 at 734 nm. Volumes of 100 µL from each tested sample were transferred into 96-well microplates and serially diluted. Then, 100 µL of ABTS•+ solution was added to each well, followed by incubation for 6 min at room temperature in the dark [29,30,31].

Absorbance was measured at 734 nm using an Epoch Microplate Spectrophotometer. TEAC values were calculated as the ratio of the IC_50_ of Trolox to that of the tested antioxidant compound [28,29,30,31].

### 2.4. Preparation of Cosmeceutical Formulations

The moisturising and anti-ageing cosmeceutical formulations investigated in this study were developed as oil-in-water (O/W) and water-in-oil (W/O) emulsion systems using three optimised cream bases, coded F1, F14, and F27. These formulation platforms were selected following extensive preformulation and optimisation studies previously conducted by our research group, including rheological characterisation, texture profile analysis, mechanical stability assessment, sensory performance evaluation, and skin tolerability testing [32,33].

Prior to active-compound incorporation, several cream bases with different emulsifying systems and formulation architectures were developed and comparatively evaluated in preliminary studies [32,33]. Based on their physicochemical and mechanical stability, rheological and textural properties, sensory performance, and preliminary cutaneous tolerability, three structurally distinct bases were selected. F1 was a water-in-oil (W/O) emulsion stabilised with methyl glucose sesquistearate, whereas F14 and F27 were oil-in-water (O/W) emulsions prepared with Ceteareth-20 and polyglyceryl-3 methylglucose distearate, respectively. Melatonin and *N*-acetylcysteine were subsequently incorporated at constant concentrations into these previously investigated bases. This design enabled the influence of vehicle architecture on melatonin permeation to be examined across different formulation platforms.

The use of formulation platforms with distinct emulsification mechanisms and microstructural organisation enabled investigation of how vehicle composition influences antioxidant activity, skin retention, permeation behaviour, and clinical anti-ageing efficacy. This approach was designed to provide a comprehensive understanding of the vehicle’s contribution to the overall performance of the developed cosmeceutical systems.

To evaluate the influence of *N*-acetylcysteine (NAC) on melatonin-based topical formulations, two formulation series were prepared. The formulations containing both melatonin and *N*-acetylcysteine were designated F1NACM, F14NACM, and F27NACM, whereas the corresponding formulations containing melatonin alone were coded F1M, F14M, and F27M. The formulations were prepared using an Unguator EMP system. All ingredients were accurately weighed. Hyaluronic acid was hydrated for 2 h in the presence of Aloe barbadensis leaf juice. The lipophilic phase was heated and fluidized separately, except for tocopherol, which was added after emulsion structuring. The hydrophilic phase was heated to a similar temperature.

The two phases were mixed for 3 min at 300 rpm, then homogenised for 3 min at 600 rpm and 3 min at 1000 rpm, all without opening the container. Tocopherol and Euxyl^®^ PE 9010 were then added, and homogenization was continued for 3 min at 1400 rpm and 3 min at 1900 rpm. Finally, melatonin and, where applicable, *N*-acetylcysteine were incorporated, followed by homogenization for 3 min at 2400 rpm.

The investigated formulations are part of a patented topical cosmeceutical platform protected under Romanian patent application no. 2025000333 (Publication No. RO 139284 A0), entitled “Highly Effective Anti-Wrinkle Cosmeceutic Product with Optimized Formulation”.

### 2.5. HPLC Method for Melatonin Quantification

Melatonin quantification was performed using a Thermo Finnigan Surveyor HPLC system equipped with a photodiode array UV-Vis detector. Chromatographic separation was carried out on a Gemini NX C18 column, 3.0 × 100 mm, with 3 µm particle size. Samples were filtered through 0.45 µm Millipore filters before injection.

The injection volume was 2 µL. The mobile phase consisted of ultrapure water and acetonitrile in a 52:48 ratio (*v*/*v*). The flow rate was 0.6 mL/min, and detection was performed at 223 nm. The system pressure was maintained at approximately 8 MPa.

A melatonin stock solution (1 mg/mL) was prepared by dissolving 100.0000 mg of melatonin in phosphate buffer (pH 7.4), adjusting the final volume to 100 mL, and applying ultrasonication until complete dissolution was achieved. For linearity assessment, seven calibration standards were prepared in the range of 0.5–50 µg/mL: 0.5, 1, 2.5, 5, 10, 25, and 50 µg/mL.

Method specificity was assessed using blank matrices without melatonin that were processed under the same conditions as the test samples. Linearity, goodness of fit, carry-over, precision, accuracy, and quantification limits were evaluated. The method was considered suitable for quantifying melatonin in the receptor medium during the permeation study. Previously reported HPLC methods for melatonin quantification in pharmaceutical and cosmetic systems were used for comparison [34,35,36,37].

### 2.6. In Vitro Permeation Study

The in vitro permeation of melatonin from the developed cosmeceutical formulations was evaluated using Franz diffusion cells fitted with Strat-M® synthetic membranes (Transdermal Diffusion Test Model, Merck Millipore, Darmstadt, Germany) [19].

The study was conducted over 24 h. The diffusion area of the Franz cell was 0.9993 cm^2^. The donor compartment contained 1 g of formulation, corresponding to 10 mg melatonin. The receptor compartment was filled with a phosphate-buffered solution (pH 7.4) and maintained at 37 ± 1 °C.

Samples of 1.6 mL were withdrawn from the receptor medium at 0.5, 1, 1.5, 2, 4, 6, 8, 10, 12, and 24 h. After each sampling, the withdrawn volume was replaced with the same volume of fresh phosphate buffer preheated to 37 ± 1 °C. The amount of melatonin permeated through the membrane was quantified using the validated HPLC method described above.

The cumulative permeation rate was calculated as:

Qn = Cn/A(2)

where Cn is the cumulative amount of melatonin permeated and A is the membrane diffusion area.

The steady-state flux (Jss, µg/h/cm^2^) was calculated from the slope of the linear portion of the permeation curve between 10 and 24 h. The steady-state flux was calculated separately for each diffusion cell from the slope of the linear portion of the cumulative permeation profile between 10, 12 and 24 h.

The present work also benefits from our previous experience in the development and validation of analytical methods for the quantitative assessment of active compounds in complex pharmaceutical systems [38], as well as in the characterisation of transdermal delivery systems and in advanced analytical approaches for drug quantification and release studies [39]. These complementary investigations contributed to the methodological framework employed in the current study and supported the accurate evaluation of melatonin stability, quantification, and permeation behavior within the developed cosmeceutical formulations. Each formulation was evaluated in three separate Franz diffusion cells using an individual Strat-M^®^ membrane for each cell. The three diffusion cells were considered independent experimental replicates. HPLC replicate injections of samples collected from the same cell were considered technical replicates.

### 2.7. Clinical Evaluation of Anti-Wrinkle Efficacy

The present study received approval from the Ethics Commission no. 2942/18.03.2024. Informed consent was obtained from all subjects involved in the study.

Potential participants underwent an initial eligibility check based on age, regular use of facial cosmetic products, and the presence of visible signs of facial ageing. Women who did not regularly use cosmetic products or who did not present the minimum required degree of periocular wrinkling were excluded at this preliminary stage. Ten potentially eligible women aged 55–65 years were subsequently invited for formal clinical screening. Following assessment of the predefined inclusion and exclusion criteria, six participants met all eligibility requirements, were enrolled, completed the 28-day study, and were included in the final analysis.

The clinical evaluation followed a participant- and evaluator-blinded design. Both formulations were placed in coded containers, and neither the participants nor the technician responsible for the clinical assessments was aware of the identity or composition of the products. The formulation codes were disclosed only after completion of the clinical evaluation and finalisation of the data analysis. The formulations were supplied in visually identical coded containers.

A participant- and evaluator-blinded split-face design was used. Each participant applied both formulations, with one coded product assigned to the right side and the other to the left side of the face. The coded containers did not disclose the identity or composition of the formulations.

#### 2.7.1. Inclusion and Exclusion Criteria

Inclusion criteria. Participants were eligible for inclusion if they were healthy female volunteers aged 55–65 years with Fitzpatrick skin phototypes II–IV, presenting crow’s-feet wrinkles of at least grade 2, low facial skin elasticity, regular use of facial cosmetic products, health insurance coverage, and written informed consent. Participants were also required to comply with the study procedures and attend all scheduled visits.

Exclusion criteria. Subjects were excluded if they had a personal or family history of atopy, known allergy to any product component, pregnancy or breastfeeding, recent use of medications or cosmetic procedures likely to interfere with study outcomes, excessive UV exposure, participation in another clinical study, or any medical condition that could compromise protocol compliance or data interpretation. Participants who failed to comply with the study protocol or who developed conditions requiring interfering treatments during the study were also excluded.

#### 2.7.2. Glogau Wrinkle Assessment

Wrinkle severity was assessed in the periocular area, which is commonly referred to as the “crow’s feet” region, using the Glogau wrinkle scale. The evaluation was performed by a trained technician under the investigator’s supervision. The Glogau scale is widely used for clinical assessment of wrinkle severity, photoaging, and the efficacy of anti-ageing treatments [40].

Clinical scoring was performed at two time points: before the first application of the tested product (D1/T0) and after 28 consecutive days of product use (D29). The anti-wrinkle effect was assessed by comparing the Glogau score obtained at D29 with the baseline score. A decrease in the Glogau score was considered indicative of improved wrinkle appearance.

#### 2.7.3. Lower Facial Ptosis Assessment

Lower facial ptosis was evaluated as an additional clinical parameter of anti-ageing efficacy. The assessment focused on the lower hemiface bilaterally, including the chin and mandibular contour. The degree of ptosis was clinically scored by the investigator using a six-grade visual scale established by the testing centre.

The ptosis scale ranged from grade 0, corresponding to the absence of ptosis and a perfectly oval chin contour, to grade 5, corresponding to severe ptosis with pronounced skin laxity. Clinical assessments were performed before the first product application (D1/T0) and after 28 consecutive days of use (D29). A reduction in ptosis score after treatment was considered indicative of improved skin firmness and lower facial contour.

### 2.8. Statistical Analysis

For the DPPH and ABTS/TEAC assays, three independent experimental runs were performed using freshly prepared solutions. Within each run, absorbance measurements were performed in technical triplicate, and the mean value of the technical replicates was used as the result of that experimental run. The reported values therefore represent the mean ± standard deviation of three independent experiments (n = 3). For the permeation study, each formulation was evaluated in three separate Franz diffusion cells, each equipped with an individual Strat-M^®^ membrane; these were treated as independent experimental replicates (n = 3). Replicate HPLC injections of the same sample were considered technical replicates and were not treated as independent observations. The clinical analysis included six independent participants (n = 6). For the antioxidant assays, statistical analyses were performed using GraphPad Prism software. Differences between the two groups were analysed using Student’s *t*-test, and *p*-values < 0.05 were considered statistically significant.

For the in vitro permeation study, statistical analysis was performed using GraphPad Prism 10.2 (Dotmatics, Boston, MA, USA). Differences among formulations were evaluated at 0.5, 12, and 24 h using Brown–Forsythe ANOVA and Welch’s ANOVA tests, followed by Dunnett’s T3 multiple comparison test. Statistical significance was expressed as follows: *p* < 0.05, *p* < 0.01, *p* < 0.001, and *p* < 0.0001.

For clinical evaluations, scores obtained at D1/T0 and D29 were compared to assess within-product efficacy, while comparisons between formulations were performed to evaluate differences in clinical performance.

## 3. Results

### 3.1. Antioxidant Activity

The chemical radical-scavenging capacity of melatonin and *N*-acetylcysteine was evaluated using the DPPH and ABTS/TEAC assays. These complementary cell-free methods provide comparative information on the reactivity of the tested compounds toward stable radical species but do not assess intracellular antioxidant activity or biological protection.

#### 3.1.1. DPPH Radical Scavenging Assay

*N*-acetylcysteine demonstrated significantly higher antioxidant activity compared to melatonin in the DPPH assay. The mean IC_50_ for *N*-acetylcysteine was 5.96 ± 1.02 µg/mL, whereas melatonin showed a markedly higher IC_50_ of 530.11 ± 48.63 µg/mL. As expected, ascorbic acid exhibited the strongest antioxidant activity, with an IC_50_ value of 0.577 ± 0.012 µg/mL (Table 1). Significant differences between groups were observed (*p* < 0.0001).

Under the cell-free conditions of the DPPH assay, *N*-acetylcysteine exhibited greater radical-scavenging activity than melatonin. This assay evaluates chemical reactivity toward the DPPH radical and does not provide information on intracellular glutathione synthesis, mitochondrial protection, endogenous antioxidant enzyme activity, or extracellular matrix preservation. Lower IC_50_ values indicate greater free-radical-scavenging activity and, therefore, stronger antioxidant potential (Figure 1).

#### 3.1.2. ABTS/TEAC Assay

**Figure 2:**
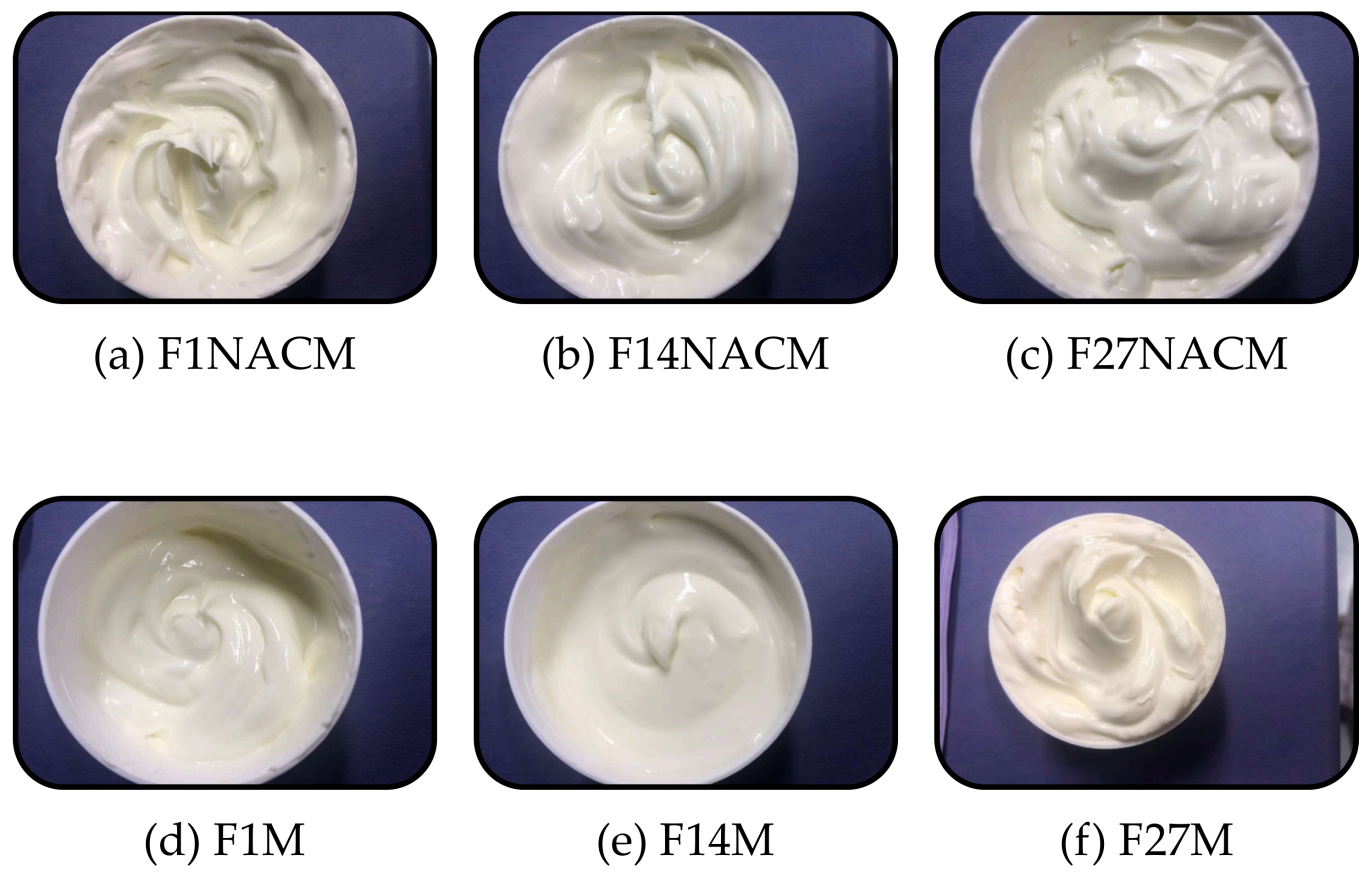
Macroscopic appearance of the developed cosmeceutical formulations: (**a**) F1NACM, (**b**) F14NACM, (**c**) F27NACM, (**d**) F1M, (**e**) F14M, and (**f**) F27M.

The ABTS assay confirmed the antioxidant activity of both tested compounds. *N*-acetylcysteine showed significantly higher TEAC values (30.02 ± 4.93 mmol/L) compared to melatonin (8.76 ± 0.54 mmol/L) (Table 2, Figure 2), corresponding to an approximately 242% higher antioxidant capacity.

Under the cell-free conditions employed in the present study, *N*-acetylcysteine exhibited greater chemical radical-scavenging capacity than melatonin in both the DPPH and ABTS/TEAC assays. This difference may be partly related to the chemical reactivity of the free thiol group of *N*-acetylcysteine. Beyond direct radical scavenging, Aldini et al. described *N*-acetylcysteine as an antioxidant and redox-modulating agent whose biological effects include support of intracellular glutathione availability [13]. Other cellular studies have also reported improvements in redox homeostasis and attenuation of oxidative stress-related damage following *N*-acetylcysteine exposure [30,31]. Similarly, although melatonin showed lower direct radical-scavenging capacity under the conditions of the present assays, previous studies have described additional biological mechanisms, including modulation of endogenous antioxidant enzymes and mitochondrial protection. These intracellular effects were not evaluated in the present investigation and are cited only to provide a literature-based biological context for the cell-free findings. The ABTS/TEAC results demonstrate differences in the chemical radical-scavenging capacity of melatonin and *N*-acetylcysteine under cell-free experimental conditions. The assay does not establish intracellular antioxidant activity or biological protection resulting from their combined use. Accordingly, no direct causal relationship can be established between the chemical radical-scavenging capacity measured in vitro and the clinical changes observed after topical application.

### 3.2. Preparation of Cosmeceutical Formulations

Following the antioxidant characterisation of melatonin and *N*-acetylcysteine, six topical cream formulations were developed to investigate the influence of different emulsifying systems on the cutaneous delivery of melatonin and its anti-ageing performance. The formulations were designed as multifunctional antioxidant-based cosmeceutical systems intended for mature (55+) skin, combining active ingredients with complementary antioxidant, moisturising, and barrier-supporting properties. In particular, the incorporation of melatonin and *N*-acetylcysteine was intended to simultaneously target direct radical scavenging, endogenous antioxidant defence mechanisms, and local oxidative stress associated with skin ageing. The composition of the investigated formulations is presented in Table 3.

### 3.3. HPLC Method Development and Validation

The optimized HPLC method enabled rapid melatonin quantification with a retention time of approximately 2.4 min and a total analysis time of 3 min. The method demonstrated excellent linearity over the concentration range of 0.5–50 µg/mL, with a coefficient of determination R^2^ of 0.9997 (Figure S1, Table 4).

The linear regression equation obtained was *y* = 93,727.905*x* + 410.27877, with a correlation coefficient *R*^2^ of 0.9999.

Carry-over was assessed by injecting a blank receptor medium immediately after the upper limit of quantification (ULOQ; 50.0 µg/mL). The acceptance criterion adopted from the ICH M10 guideline required the analyte response in the blank following the ULOQ to be no greater than 20% of the analyte response at the lower limit of quantification (LLOQ) [41]. The observed response was 18.89 ± 1.07% of the response at the LLOQ and therefore met the predefined acceptance criterion. As no internal standard was used in the present method, the corresponding ICH acceptance criterion for the internal-standard response was not applicable [41].

The developed HPLC-PDA method demonstrated adequate selectivity, with no interfering peaks detected in blank samples. Excellent linearity was obtained within the concentration range of 0.5–50.0 µg/mL (R^2^ = 0.9997), while intra-day and inter-day precision remained below 5%. Carry-over at the highest validation concentration (50.0 µg/mL) was below the predefined acceptance criterion, indicating that the method is suitable for the quantitative determination of melatonin in skin permeation experiments.

### 3.4. In Vitro Permeation Study

The permeation profiles of melatonin varied significantly depending on formulation composition. After 24 h, the cumulative amount of permeated melatonin ranged from 1.62 ± 0.34% for F1M to 20.87 ± 0.67% for F14NACM.

Among all tested formulations, F14NACM exhibited the highest permeation capacity, whereas F1M showed the lowest. Statistical analysis revealed significant differences among formulations at 12 and 24 h (*p* < 0.05). These results demonstrate formulation-dependent differences in melatonin permeation across the Strat-M^®^ membrane.

The cumulative amount of melatonin permeated after 24 h (Q_24_h) ranged from 98.79 ± 25.64 µg/cm^2^ for F1M to 1181.92 ± 49.30 µg/cm^2^ for F14NACM. The steady-state flux ranged from 1.62 ± 0.66 µg·h^−1^·cm^−2^ for F1M to 39.51 ± 2.94 µg·h^−1^·cm^−2^ for F14NACM (Table 5).

**Figure 3:**
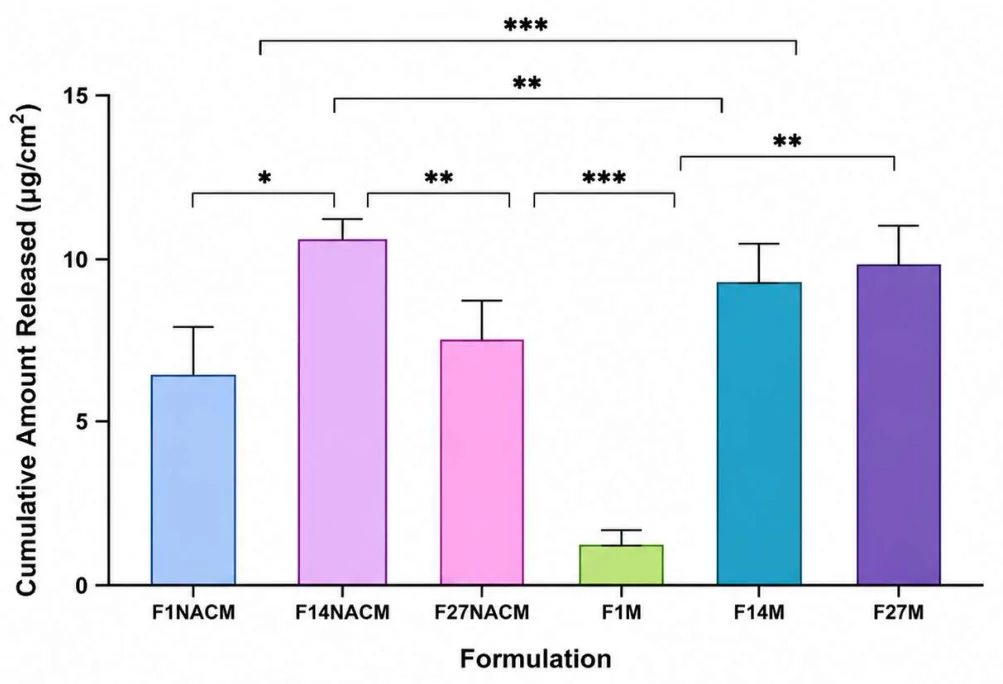
Comparison of the cumulative amount of melatonin permeated through the Strat-M® membrane after 12 h from the evaluated formulations (F1NACM, F14NACM, F27NACM, F1M, F14M, and F27M). Data are presented as mean ± SD (*n* = 3). Statistical differences between groups were evaluated using Dunnett’s T3 multiple-comparisons test: * *p* < 0.05, ** *p* < 0.01, and *** *p* < 0.001.

**Figure 4:**
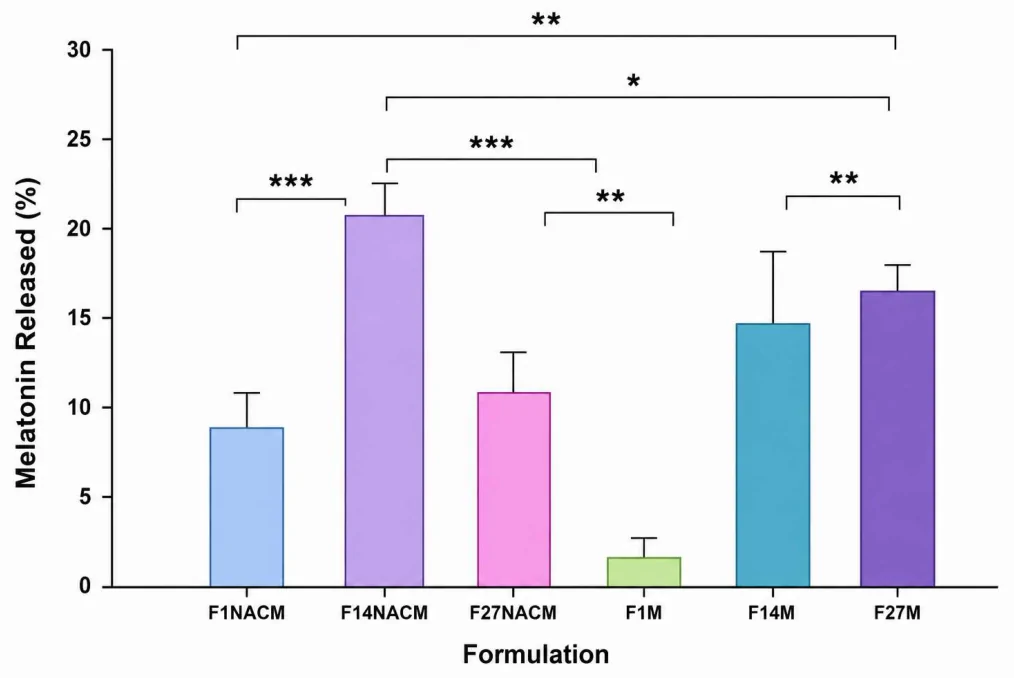
Percentage of melatonin released after 24 h from the evaluated formulations (F1NACM, F14NACM, F27NACM, F1M, F14M, and F27M). Data are presented as mean ± SD (*n* = 3). Statistical differences between groups were evaluated using Dunnett’s T3 multiple-comparisons test: * *p* < 0.05, ** *p* < 0.01, and *** *p* < 0.001.

Figure 3 and Figure 4 show that melatonin permeation differed among the six formulations. In some formulation bases, the presence of *N*-acetylcysteine was associated with a higher cumulative amount of melatonin in the receptor medium. However, the present experimental design does not establish the mechanism underlying these differences or demonstrate increased availability within specific skin layers.

The results confirm that melatonin permeation across the synthetic membrane varied according to formulation composition. Lower melatonin recovery in the receptor compartment cannot be interpreted as evidence of retention within the skin, because Strat-M^®^ does not contain anatomical skin compartments and the amount remaining in the membrane or donor formulation was not separately quantified.

### 3.5. Clinical Evaluation of Anti-Ageing Efficacy

#### 3.5.1. Glogau Wrinkle Assessment

After 28 consecutive days of product application, both F1NACM and F27NACM formulations significantly reduced the Glogau wrinkle score in the periocular area compared with baseline (Table 6 and Table 7).

For F1NACM, the mean Glogau score decreased from 2.33 ± 0.52 at baseline to 1.50 ± 0.55 after treatment, corresponding to a 36.11% reduction (*p* = 0.002). Similarly, F27NACM reduced the wrinkle score by 30.56% (*p* = 0.013).

No statistically significant differences were observed between the two formulations regarding wrinkle score reduction (Table 7).

After 28 consecutive days of product application, the periocular wrinkle clinical score showed a significant decrease at Day 29 (D29) compared with baseline (D1) for both tested formulations (F1NACM and F27NACM) (Table 8). The F1NACM formulation, containing a W/O emulsifying base, improved the periocular wrinkle condition in 83.3% of the subjects. In contrast, the F27NACM formulation, containing an O/W emulsifying base, improved the periocular wrinkle condition in 66.7% of the subjects.

The clinical wrinkle score in the periocular “crow’s feet” area did not show any significant differences between the two formulations at D29 compared with baseline (D1). From a statistical perspective, the anti-wrinkle effect, evaluated using the Glogau score before application and after 28 days of product use, did not show a statistically significant difference between the two tested products, F1NACM and F27NACM.

#### 3.5.2. Lower Facial Ptosis Assessment

Both tested formulations significantly improved lower facial ptosis after 28 days of application. The mean ptosis score decreased from 3.67 ± 0.52 at baseline to 3.17 ± 0.75 after treatment for both formulations, corresponding to a reduction of approximately 13.89%.

The decrease in ptosis scores was statistically significant for both F1NACM and F27NACM (*p* = 0.038). However, no significant differences were observed between the two formulations.

Individual results were expressed as absolute values for each experimental timepoint, as differences (Δ), and as percentage variation relative to baseline values (Table 9).

**Figure 5:**
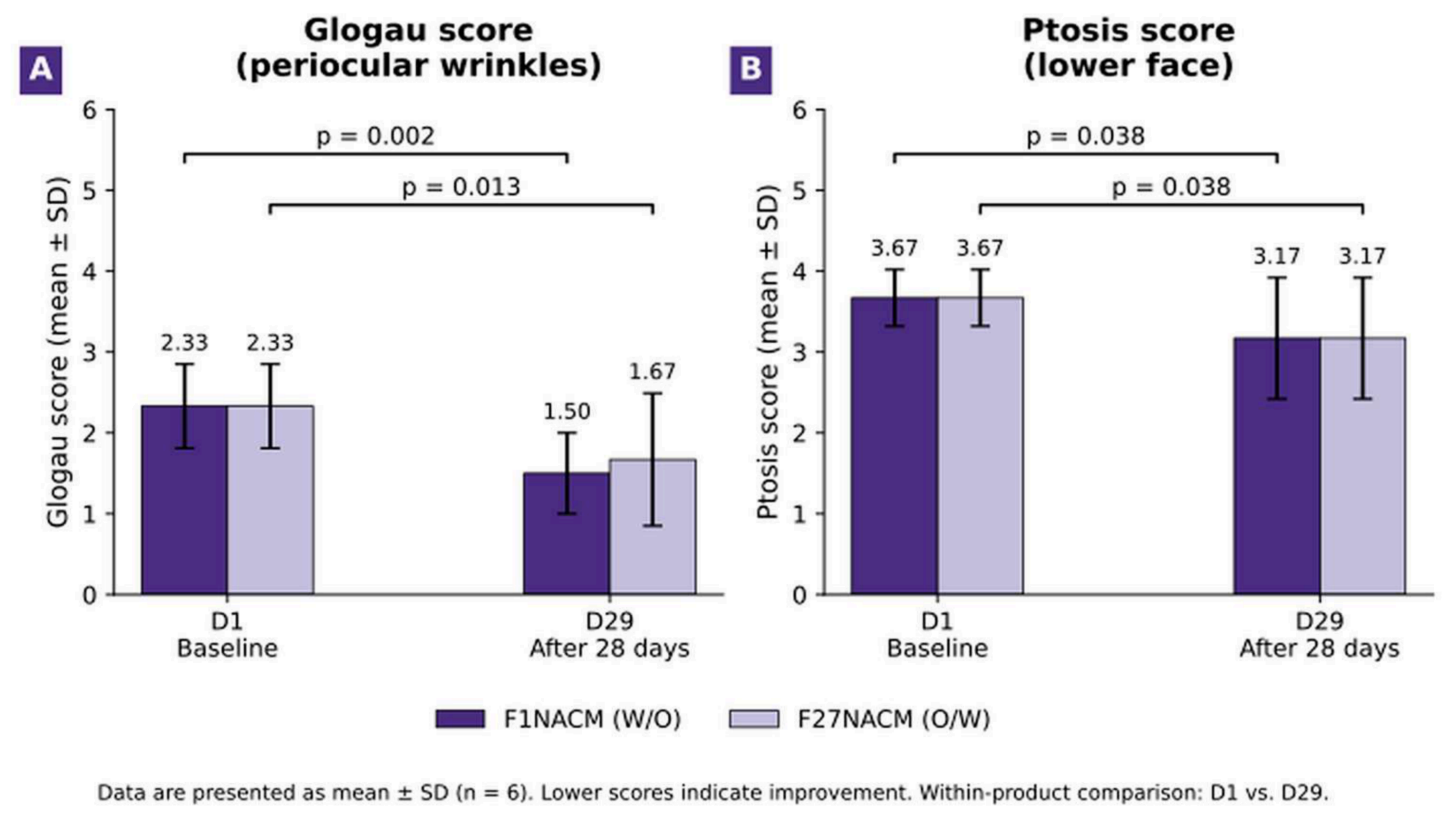
Clinical evaluation of anti-ageing efficacy after 28 days of treatment. (**A**) Changes in Glogau score in the periocular “crow’s feet” area following treatment with F1NACM and F27NACM formulations. (**B**) Changes in lower facial ptosis score after 28 days of product application. Data are presented as mean ± standard deviation (SD) (n = 6). Significant reductions compared with baseline (D1) were observed for both formulations. For the Glogau score, the reductions were significant for F1NACM (*p* = 0.002) and F27NACM (*p* = 0.013). For the ptosis score, significant improvements were observed for both formulations (*p* = 0.038). No statistically significant differences were detected between formulations for either clinical endpoint.

The mean lower facial ptosis score decreased by 13.89% after 28 days for both formulations. This change should be interpreted as a preliminary within-group clinical observation. Based on the ptosis score, no statistically significant differences were observed between the two tested formulations, F1NACM and F27NACM (Figure 5).

Both formulations were associated with reductions in Glogau wrinkle and lower facial ptosis scores after 28 days. F1NACM produced a numerically greater reduction in the Glogau score, although the difference between formulations was not statistically significant. Given the small sample size and the absence of a vehicle-control group, these results should be regarded as preliminary and do not establish comparative or definitive clinical efficacy.

## 4. Discussion

The present study evaluated topical formulations containing melatonin and *N*-acetylcysteine by combining antioxidant assays, in vitro membrane-permeation testing, and a preliminary clinical assessment. Melatonin and *N*-acetylcysteine have complementary antioxidant properties reported in the literature; however, their interaction and biological mechanisms were not directly investigated in the present study.

Oxidative stress is considered one of the main mechanisms underlying skin ageing, contributing to mitochondrial dysfunction, collagen degradation, inflammation, and wrinkle formation. In this context, melatonin has attracted increasing attention due to its multifunctional antioxidant activity, including direct free radical scavenging, mitochondrial protection, stimulation of endogenous antioxidant enzymes, and reductions in reactive oxygen species generation.

Several PubMed-indexed studies have investigated the role of topical melatonin in skin aging. Milani and collaborators demonstrated that melatonin-based day and night creams significantly improved skin hydration, tonicity, and roughness in women with facial ageing after topical application. Their study also reported a significant reduction in crow’s feet wrinkles and improved skin profilometry after three months of treatment [42].

Similarly, Day et al. reported that topical melatonin reduced markers of oxidative stress and improved visible signs of skin ageing through its antioxidant and mitochondrial-protective effects [43].

Kleszczynski and collaborators further emphasised that melatonin acts as an anti-ageing molecule at the skin level by protecting mitochondria, reducing oxidative stress, and supporting cutaneous homeostasis [44].

More recently, Greco and collaborators reviewed clinical studies involving topical melatonin and concluded that melatonin-based formulations exert photoprotective, antioxidant, and anti-ageing effects with favourable tolerability profiles [45,46].

The findings of the present study are consistent with previously published data, as both formulations investigated significantly reduced Glogau wrinkle scores and facial ptosis after 28 days of application. However, the innovative aspect of the present work lies in the association of melatonin with *N*-acetylcysteine, a combination that has been insufficiently explored in topical anti-ageing products currently available.

*N*-acetylcysteine is recognised for its strong antioxidant and anti-inflammatory activity, primarily through glutathione replenishment and the reduction in oxidative stress. Adil and collaborators highlighted the dermatological relevance of *N*-acetylcysteine for its antioxidant effects and potential role in modulating inflammatory skin disorders [47].

Janeczek and collaborators also discussed the therapeutic potential of *N*-acetylcysteine in dermatology, particularly in conditions associated with oxidative stress and impaired skin barrier function [22].

Although topical *N*-acetylcysteine formulations have primarily been investigated for wound healing and inflammatory dermatoses, studies specifically targeting anti-ageing applications remain limited. Tsai and collaborators demonstrated that topical *N*-acetylcysteine accelerated tissue regeneration and improved wound healing through antioxidant and anti-inflammatory mechanisms [48]. The mitochondrial, glutathione-related, anti-inflammatory, extracellular matrix, and barrier-related effects discussed above have been reported in previous experimental or clinical studies. The present investigation did not assess mitochondrial function, intracellular glutathione, oxidative stress biomarkers, inflammatory mediators, collagen or elastin metabolism, or skin-barrier parameters. Accordingly, these mechanisms should be regarded as literature-based explanations supporting the formulation rationale rather than mechanisms demonstrated by the current results. Compared with previously investigated topical melatonin systems, the present formulations additionally contained *N*-acetylcysteine and exhibited formulation-dependent melatonin permeation across the Strat-M^®^ membrane. Whether this combination provides additive or synergistic biological effects remains to be established [49].

The observed differences demonstrate that vehicle composition affects melatonin transport across the Strat-M^®^ membrane. Nevertheless, the study did not determine the partition coefficient of melatonin at the stratum corneum, its distribution among skin layers, or its local bioavailability. Strat-M^®^ is suitable for comparative permeation screening but does not permit direct assessment of cutaneous deposition. Future studies using excised human or porcine skin, followed by tape stripping and separate extraction of the stratum corneum, viable epidermis, dermis, and receptor compartment, are required to establish whether any of the formulations favour superficial cutaneous retention. The concept proposed in the present work aligns with the modern evolution of cosmeceuticals, which are considered hybrid products situated at the interface between cosmetics and pharmaceuticals. Unlike conventional cosmetic products focused mainly on hydration or sensory properties, cosmeceuticals contain biologically active compounds capable of exerting measurable physiological effects at the skin level.

The DPPH and ABTS/TEAC assays provide evidence of the antioxidant capacity of the individual compounds under cell-free experimental conditions. The mitochondrial, anti-inflammatory, glutathione-related, and extracellular matrix mechanisms discussed above are supported by the previous literature but were not directly assessed in the present study. No cellular, biochemical, histological, or molecular biomarkers were measured. Therefore, these mechanisms should be considered a biological rationale for further investigation rather than demonstrated explanations for the observed clinical changes [46,49]. The preliminary clinical findings indicate reductions in wrinkle and ptosis scores after 28 days of product use. However, the study did not include a vehicle-control, untreated-control, or active-comparator group. Moreover, the formulations contained other ingredients with potential moisturising, antioxidant, and skin-conditioning effects, including hyaluronic acid, Aloe barbadensis, Acmella extract, and α-tocopherol. Consequently, the observed changes cannot be attributed specifically to melatonin, *N*-acetylcysteine, or their combination. Future clinical investigations should include a larger and more diverse population and a randomised, blinded, vehicle-controlled design. The control formulation should have the same cream base as the investigational product but should not contain melatonin or *N*-acetylcysteine. Additional comparison groups containing melatonin alone and *N*-acetylcysteine alone would help determine the individual contribution of each active ingredient and establish whether their combined use produces additive or synergistic effects. Comparison with an appropriate commercially available antioxidant or anti-ageing product would also improve the clinical relevance of the findings. If a marketed formulation containing both melatonin and *N*-acetylcysteine becomes available, its inclusion as an active comparator would be particularly informative. Although reductions in wrinkle and ptosis scores were observed after 28 days, the present data do not establish the biological mechanisms responsible for these changes. In addition, because the formulations contained several potentially active ingredients, the clinical observations cannot be attributed specifically to the mitochondrial or antioxidant effects of melatonin, the glutathione-related activity of *N*-acetylcysteine, or their combination.

This programme included several preliminary investigations, some of which have been published, addressing formulation stability, compatibility, sensory characteristics, skin tolerability, and the topical use of melatonin and *N*-acetylcysteine. Other preformulation findings have not yet been published. During formulation development, the concentrations of the active ingredients were progressively increased from lower initial levels and evaluated in relation to formulation performance, tolerability, and preliminary efficacy.

This broader research programme included published and unpublished preformulation studies addressing formulation stability, compatibility, sensory performance, and skin tolerability. In a previous study, multi-active formulations containing *N*-acetylcysteine demonstrated suitable physical stability, favourable sensory characteristics, and good short-term cutaneous tolerability [32].

The selected *N*-acetylcysteine concentration reflected a balance between functional performance and sensory acceptability. Although concentrations above 5% were considered during preliminary development, they produced an intense and persistent sulfurous odour that could not be acceptably masked by fragrance and would have substantially compromised the sensory characteristics of a facial product and adherence to repeated use. This limitation is consistent with previous reports describing objectionable odour and irritation as practical barriers to the topical use of *N*-acetylcysteine. Dermatological formulations containing 10% *N*-acetylcysteine, frequently combined with 5% urea, have been reported in the treatment of ichthyosis, whereas a formulation containing 5% *N*-acetylcysteine and 5% urea has also been described [50,51,52]. However, these therapeutic preparations were developed for disorders of keratinisation and their concentrations cannot be directly extrapolated to a leave-on anti-ageing facial cosmeceutical.

The concentration of melatonin was selected with consideration of published topical studies. Previous investigations evaluated melatonin at 0.01% in a cream and at 0.01–0.03% in alcoholic solutions in a percutaneous penetration study, while concentrations of 0.5%, 2.5%, and 12.5% were examined in a dose-dependent photoprotection study [53,54]. These markedly different concentrations reflect differences in study objectives, formulation type, application area, and exposure conditions and should not be interpreted as generally applicable cosmetic concentration limits.

No substance-specific maximum concentrations for melatonin or *N*-acetylcysteine were identified in the relevant restricted-substance annexes of Regulation (EC) No. 1223/2009. Nevertheless, the absence of a harmonised concentration limit does not, by itself, establish the safety of a particular use level. The selected concentrations must be considered in relation to the complete formulation, intended site and frequency of application, physicochemical stability, cutaneous exposure, and toxicological profile. Future development should therefore include a product-specific assessment of potential systemic exposure and completion of a Cosmetic Product Safety Report before commercialisation.

## 5. Conclusions

*N*-acetylcysteine exhibited greater chemical radical-scavenging capacity than melatonin in the DPPH and ABTS/TEAC assays, while the six tested cream formulations showed distinct melatonin permeation profiles across the Strat-M^®^ membrane. These findings demonstrate that formulation composition influences in vitro melatonin permeation but do not establish retention or deposition within individual skin layers. Given the small sample size, short treatment period, absence of a vehicle-control group, and presence of other potentially active ingredients, these results should be interpreted as preliminary clinical observations rather than definitive evidence of efficacy attributable to melatonin and *N*-acetylcysteine. Reductions in Glogau wrinkle and lower facial ptosis scores were observed after 28 days of application of F1NACM and F27NACM. However, because the clinical evaluation included only six participants and did not include a vehicle-control or active-comparator group, these changes cannot be attributed specifically to melatonin, *N*-acetylcysteine, or their combination. The formulations contained several other ingredients that may have contributed to the observed effects. Therefore, the results should be regarded as preliminary evidence of the overall clinical performance of the complete formulations rather than definitive evidence of efficacy of the two selected antioxidants. The cell-free antioxidant results should not be interpreted as direct evidence of intracellular antioxidant activity or as confirmation of the biological mechanism underlying the preliminary clinical observations.

Further studies using biological skin models, compartment-specific quantification, objective instrumental measurements, and larger randomised controlled clinical cohorts are required to determine cutaneous deposition, clarify the contribution of each active ingredient, and confirm the clinical relevance of these formulations.

## 6. Limitations

Several limitations should be considered when interpreting the present findings. The clinical evaluation included only six participants, was conducted over 28 days, and did not include randomisation, a vehicle-control group, an untreated control, or an active comparator. The clinical outcomes were based primarily on visual scoring scales, without complementary instrumental measurements such as wrinkle profilometry, cutometry, corneometry, transepidermal water loss, or high-resolution skin imaging. Accordingly, the clinical findings should be regarded as exploratory.

The formulations also contained other ingredients with potential moisturising, antioxidant, and skin-conditioning effects, including hyaluronic acid, *Aloe barbadensis*, *Acmella* extract, and α-tocopherol. Therefore, the individual contributions of melatonin and *N*-acetylcysteine and any additive or synergistic interaction between them could not be determined. In addition, the antioxidant assays were cell-free chemical tests and do not directly demonstrate antioxidant activity within human skin. Cellular or tissue-based studies measuring intracellular reactive oxygen species, glutathione status, antioxidant enzyme activity, and extracellular matrix biomarkers are required to investigate the biological relevance of the chemical radical-scavenging results. The mitochondrial, anti-inflammatory, glutathione-related, and extracellular matrix mechanisms discussed in the manuscript were derived from previously published evidence and were not directly investigated. No cellular, biochemical, histological, or molecular analyses were performed to confirm these mechanisms.

The permeation experiments were performed using Strat-M^®^, a synthetic membrane intended for comparative permeation screening. This model does not reproduce the anatomical compartments, metabolic activity, or interindividual variability of human skin and cannot demonstrate cutaneous retention or deposition. Moreover, a complete mass-balance assessment, including the amounts remaining in the donor formulation and within the membrane, was not performed. The results should therefore be interpreted exclusively as comparative in vitro membrane-permeation data. Studies using excised human or porcine skin, tape stripping, and separate quantification in the stratum corneum, viable epidermis, dermis, and receptor compartment are required to characterize the cutaneous distribution of melatonin. The absence of a vehicle-control formulation represents an important limitation. Both investigated formulations had complex compositions and contained, in addition to melatonin and *N*-acetylcysteine, other ingredients with potential moisturising, antioxidant, film-forming, or skin-conditioning effects, including hyaluronic acid, *Aloe barbadensis*, *Acmella* extract, and α-tocopherol. Consequently, the observed changes in wrinkle and ptosis scores cannot be attributed specifically to melatonin, *N*-acetylcysteine, or their combination. A vehicle-control cream without either active compound, together with formulations containing each antioxidant separately, would be required to distinguish the effects of the formulation base from those of the individual active ingredients.

The absence of an active-free vehicle control represents a major limitation. Although the split-face design enabled a paired comparison of two formulations containing identical concentrations of melatonin and *N*-acetylcysteine, it did not permit the observed changes to be attributed specifically to either active compound. A subsequent ethically approved controlled study, incorporating a vehicle comparator, skin-elasticity measurements, and wrinkle profilometry using silicone replicas, is currently underway.

## 7. Future Perspectives

Future investigations will focus on quantifying *N*-acetylcysteine (NAC) permeation across the Strat-M^®^ membrane using a validated HPLC method and on elucidating the relationship between NAC and melatonin permeation profiles in the developed formulations. Pearson’s and Spearman’s correlation analyses will be employed to assess potential associations between the transmembrane transport of both active compounds. Furthermore, future studies in a larger cohort of volunteers are envisaged to further substantiate the anti-ageing efficacy of these antioxidant formulations. In addition to clinical assessments based on the Glogau classification and lower-face ptosis scores, objective instrumental evaluations, including wrinkle profilometry and skin hydration measurements, may be incorporated to provide a more comprehensive characterisation of the anti-wrinkle and skin-conditioning effects of the developed formulations. Future studies should evaluate these formulations in larger, randomised, and appropriately blinded clinical cohorts. A vehicle-control formulation without melatonin and *N*-acetylcysteine should be included, together with formulations containing melatonin alone, *N*-acetylcysteine alone, and their combination. This design would allow the effects of the vehicle and each active ingredient to be distinguished and would permit formal evaluation of potential additive or synergistic interactions. An appropriate commercially available anti-ageing product should also be included as an active comparator. Objective instrumental measurements, including wrinkle profilometry, cutometry, corneometry, transepidermal water loss, and high-resolution skin imaging, should complement clinical scoring.

## 8. Patents

The formulations evaluated in this study are protected under Romanian patent application no. 2025000333 (Publication No. RO 139284 A0), entitled “Highly Effective Anti-Wrinkle Cosmeceutic Product with Optimized Formulation”, published on 30 December 2025. The patent covers the composition and use of optimised topical anti-ageing cosmeceutical formulations.

## Figures and Tables

**Table 1:** Antioxidant activity of the analysed compounds expressed as IC_50_ values determined using the DPPH radical-scavenging assay.

Sample	IC_50_ (µg/mL)
*N*-acetylcysteine	5.96 ± 1.02
Melatonin	530.11 ± 48.63
Ascorbic acid	0.577 ± 0.012

**Table 2:** Antioxidant activity of *N*-acetylcysteine and melatonin against the ABTS•+ radical, expressed as Trolox equivalent antioxidant capacity (TEAC).

Sample	TEAC (mmol/L)
*N*-acetylcysteine	30.02 ± 4.93
Melatonin	8.76 ± 0.54

Data are presented as mean ± standard deviation (SD) of three independent determinations (n = 3). *N*-acetylcysteine exhibited significantly higher TEAC values than melatonin (Student’s *t*-test, *p* < 0.01).

**Table 3:** Composition of the cosmeceutical formulations containing melatonin and *N*-acetylcysteine.

Ingredient (INCI)	F1NACM (%)	F14NACM (%)	F27NACM (%)	F1M (%)	F14M (%)	F27M (%)
Melatonin	1	1	1	1	1	1
*N*-acetylcysteine	5	5	5	–	–	–
Arginine HCL	1	1	1	1	1	1
Blainvillea acmella flower extract	1	1	1	1	1	1
Tocopherol	1	1	1	1	1	1
Hyaluronic acid LMW	1.4	1.4	1.4	1.4	1.4	1.4
Aloe barbadensis leaf juice	20	20	20	20	20	20
Achillea millefolium oil	7	7	7	7	7	7
Cocos nucifera oil	7	7	7	7	7	7
Argania spinosa kernel oil	7	7	7	7	7	7
Centaurea cyanus flower water	39.6	39.6	38.1	39.6	39.6	38.1
Euxyl^®^ PE 9010	1	1	1	1	1	1
Methyl glucose sesquistearate	4	–	–	4	–	–
Stearic acid	4	–	3	4	–	3
Ceteareth^®^ 20	–	4	–	–	4	–
Cetyl alcohol	–	6	–	–	6	–
Polyglyceryl-3 methylglucose distearate	–	–	3.5	–	–	3.5
Cetyl palmitate	–	–	3	–	–	3

**Table 4:** Analytical performance parameters of the developed HPLC method.

Parameter	Result
Detection wavelength	223 nm
Mobile phase composition	Water:acetonitrile (52:48, *v*/*v*)
Retention time (tR)	2.4 min
Total analysis time	3.0 min
Linearity range	0.5–50.0 µg/mL
Regression equation	y = 93,727.905x + 410.279
Coefficient of determination (R^2^)	0.9997
Lower validation concentration	0.5 µg/mL
Intermediate validation concentration	5.0 µg/mL
Upper validation concentration	50.0 µg/mL
Carry-over at 50.0 µg/mL	18.89 ± 1.07%
Intra-day variability	<5%
Inter-day variability	<5%

**Table 5:** The parameters used to evaluate the permeation properties.

Formulation	Q_24_h (µg/cm^2^)	J_ss (µg·h^−1^·cm^−2^)
F1NACM	499.25 ± 66.63	12.37 ± 0.48
F14NACM	1181.92 ± 49.30	39.51 ± 2.94
F27NACM	620.41 ± 4.49	18.59 ± 1.17
F1M	98.79 ± 25.64	1.62 ± 0.66
F14M	844.74 ± 189.66	23.92 ± 19.01
F27M	939.01 ± 74.12	27.76 ± 4.19

Q_24_h, cumulative amount of melatonin permeated per unit diffusion area after 24 h; J_ss, steady-state flux. Values are expressed as mean ± SD from three independent Franz diffusion cells per formulation (n = 3), each equipped with an individual Strat-M^®^ membrane. Q_24_h and J_ss were calculated separately for each diffusion cell before calculation of the group mean and standard deviation. J_ss was determined from the slope of the linear portion of the cumulative permeation profile between 10 and 24 h.

**Table 6:** Clinical evaluation of the Glogau score before and after 28 days of application of the investigational products.

Parameter	Timepoint	Product	n	Mean ± SD	Median	Min–Max
Glogau score	Baseline (D1)	F1NACM	6	2.33 ± 0.52	2.0	2.00–3.00
		F27NACM	6	2.33 ± 0.52	2.0	2.00–3.00
	Day 29 (D29)	F1NACM	6	1.50 ± 0.55	1.5	1.00–2.00
		F27NACM	6	1.67 ± 0.82	1.5	1.00–3.00
	Δ (D29–D1)	F1NACM	6	−0.83 ± 0.41	−1.00	−1.00–0.00
		F27NACM	6	−0.67 ± 0.52	−1.00	−1.00–0.00
	Percentage change (%)	F1NACM	6	−36.11 ± 19.48	−41.67	−50.00–0.00
		F27NACM	6	−30.56 ± 24.53	−41.67	−50.00–0.00

**Table 7:** Statistical comparison of Glogau scores between baseline and Day 29.

Parameter	Product	Comparison	*p*-Value	Significance
Glogau score	F1NACM	D1 vs. D29	0.002	Significant
	F27NACM	D1 vs. D29	0.013	Significant

Data are presented as mean ± SD. Statistical significance was considered at *p* < 0.05.

**Table 8:** Comparison of Glogau score values between formulations at different experimental timepoints.

Parameter	Experimental Timepoint	*p*-Value	Statistical Interpretation
Glogau score	D1	1.000	Not statistically significant
	D29	0.687	Not statistically significant
	Δ (D29–D1)	0.549	Not statistically significant
	Percentage change (D29 vs. D1)	0.673	Not statistically significant

Statistical significance was considered at *p* < 0.05.

**Table 9:** Evaluation of anti-wrinkle efficacy using the clinical ptosis score before and after 28 days of application of the investigational cosmetic product.

Parameter	Timepoint	Product	n	Mean ± SD	Median	Min–Max
Ptosis score	Baseline (D1)	F1NACM	6	3.67 ± 0.52	4.00	3.00–4.00
		F27NACM	6	3.67 ± 0.52	4.00	3.00–4.00
	Day 29 (D29)	F1NACM	6	3.17 ± 0.75	3.00	2.00–4.00
		F27NACM	6	3.17 ± 0.75	3.00	2.00–4.00
	Δ (D29–D1)	F1NACM	6	−0.50 ± 0.55	−0.50	−1.00–0.00
		F27NACM	6	−0.50 ± 0.55	−0.50	−1.00–0.00
	Percentage change (%)	F1NACM	6	−13.89 ± 15.52	−12.50	−33.33–0.00
		F27NACM	6	−13.89 ± 15.52	−12.50	−33.33–0.00

Percentage variation was calculated relative to baseline values according to the following equation: Δ% = [(Ti − T0) × 100]/T0. Data are presented as mean ± standard deviation (SD).

## Data Availability

All data are available within the article.

## Supplementary Materials

The following supporting information can be downloaded at: https://www.mdpi.com/article/10.3390/antiox15080991/s1, Figure S1. Calibration curve showing the relationship between concentration (µg/mL) and chromatographic peak area (A.U.).
