# Supplementary material for: A New Cosmeceutical Approach for Mature Skin: Antioxidant Activity, In Vitro Permeation, and Preliminary Clinical Efficacy of Melatonin–N-Acetylcysteine Creams
Source: Antioxidants (Basel). 2026 Aug 10;15(8):991. doi: 10.3390/antiox15080991 (PMC13509359; doi:10.3390/antiox15080991)
Supplement: Supplementary file 1 [file antioxidants-15-00991-s001.zip › antioxidants-4428971-supplementary.pdf]

Article

A New Cosmeceutical Paradigm for Mature Skin: Insights into the Antioxidant Activity, Skin Retention, and Clinical Efficacy of Melatonin–N-Acetylcysteine Creams

Magdalena Bîrsan[1], Ruxandra Ștefănescu[2], Daniela-Lucia Muntean[3], Robert-Alexandru Vlad [4*], Alin-Viorel Focșa[1], Cristina-Elena Iancu[1], Șadiye-Ioana Scripcariu[1*]and Adriana Ciurba[4]

[1] Grigore T. Popa University of Medicine and Pharmacy Iasi, 16 Universitatii Street, 700115, Iasi, Romania, M.B., A-V.F., C-E.I., S-I.S.

[2] Department of Pharmacognosy and Phytotherapy, Faculty of Pharmacy, "George Emil Palade" University of Medicine, Pharmacy, Sciences and Technology of Târgu Mureș, 38 Gheorghe Marinescu Street, 540139 Târgu Mureș, Romania, R.S.

[3] Department of Analytical Chemistry, George Emil Palade University of Medicine, Pharmacy, Science and Technology of Targu Mures, 540142 Mures, Romania, D-L M.

[4] Pharmaceutical Technology and Cosmetology Department, Faculty of Pharmacy, George Emil Palade University of Medicine, Pharmacy, Science and Technology of Targu Mures, 38th Gheorghe Marinescu Street, 540142 Targu Mures, Romania; R.-A.V.,A.C.

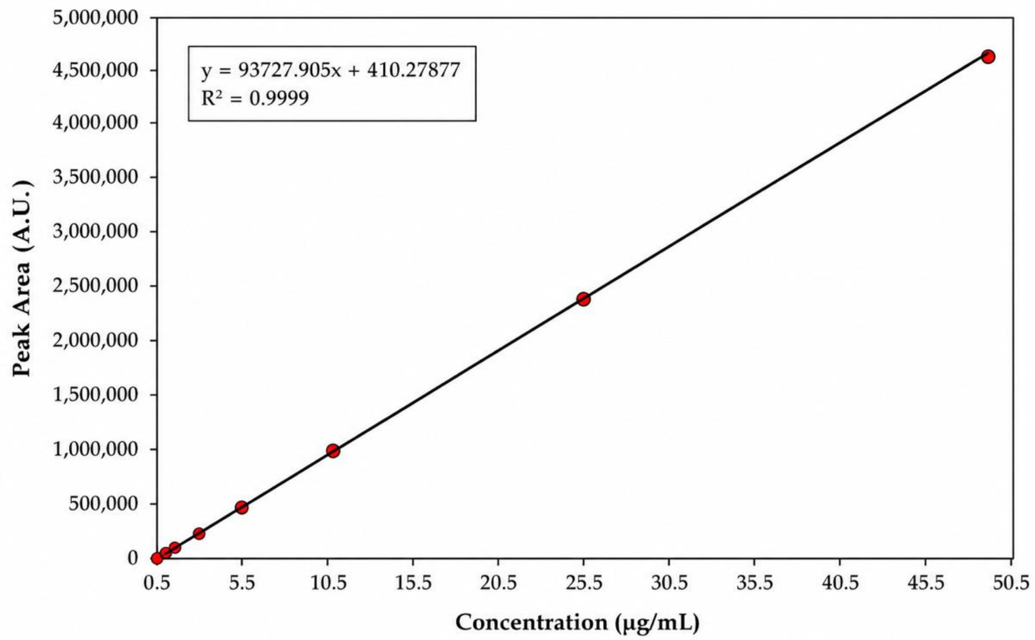

Figure S1. Calibration curve showing the relationship between concentration (μg/mL) and chromatographic peak area (A.U.).
